# Vegetation structure drives mosquito community composition in UK’s largest managed lowland wetland

**DOI:** 10.1186/s13071-024-06280-y

**Published:** 2024-05-06

**Authors:** Daniel C. Smith, Stefanie M. Schäfer, Nick Golding, Miles A. Nunn, Steven M. White, Amanda Callaghan, Bethan V. Purse

**Affiliations:** 1 UK Centre for Ecology and Hydrology, MacLean Building, Wallingford, OX10 8BB UK; 2 School of Biological Sciences, University of Reading, Whiteknights, Reading, RG6 2AJ UK

**Keywords:** Mosquito, Wetlands, Management, Disease, Community ecology

## Abstract

**Purpose:**

The rising burden of mosquito-borne diseases in Europe extends beyond urban areas, encompassing rural and semi-urban regions near managed and natural wetlands evidenced by recent outbreaks of Usutu and West Nile viruses. While wetland management policies focus on biodiversity and ecosystem services, few studies explore the impact on mosquito vectors.

**Methods:**

Our research addresses this gap, examining juvenile mosquito and aquatic predator communities in 67 ditch sites within a South England coastal marsh subjected to different wetland management tiers. Using joint distribution models, we analyse how mosquito communities respond to abiotic and biotic factors influenced by wetland management.

**Results:**

Of the 12 mosquito species identified, *Culiseta annulata* (Usutu virus vector) and *Culex pipiens* (Usutu and West Nile virus vector) constitute 47% of 6825 larval mosquitoes. Abundant predators include Coleoptera (water beetles) adults, Corixidae (water boatmen) and Zygoptera (Damselfy) larvae. Models reveal that tier 3 management sites (higher winter water levels, lower agricultural intensity) associated with shade and less floating vegetation are preferred by specific mosquito species. All mosquito species except *Anopheles maculipennis* s.l., are negatively impacted by potential predators. *Culiseta annulata* shows positive associations with shaded and turbid water, contrary to preferences of Corixidae predators.

**Conclusions:**

Tier 3 areas managed for biodiversity, characterised by higher seasonal water levels and reduced livestock grazing intensity, provide favourable habitats for key mosquito species that are known vectors of arboviruses, such as Usutu and West Nile. Our findings emphasise the impact of biodiversity-focused wetland management, altering mosquito breeding site vegetation to enhance vector suitability. Further exploration of these trade-offs is crucial for comprehending the broader implications of wetland management.

**Supplementary Information:**

The online version contains supplementary material available at 10.1186/s13071-024-06280-y.

## Background

The burden and risk of mosquito-borne diseases (MBDs) is increasing across Europe, not only in urban areas driven by invasive Aedes mosquitoes (e.g. Dengue, Chikungunya, Zika, [54]), but also by native species in more rural or peri-urban areas, at the interfaces between human habitation, agriculture and natural ecosystems (e.g. West Nile virus, Usutu, Sindbis, [10, 15]). These changes in risk are attributed to multiple interacting global drivers including climate change [9], increased trade and travel [6, 46] and land use change, including agricultural intensification and urbanisation [36, 57, 78]. At local scales, human activities in areas with long-standing mosquito presence can be a driver of MBD risk by increasing potential contact rates between people and competent vectors [47]. Man-made habitat modification that leads to shifts in abundance and species composition of mosquito populations can also alter the interaction dynamics between mosquitoes, humans and animal reservoir hosts, increasing the relative risk of zoonotic disease spillover [57].

In parallel, there is an increased policy focus on managing natural ecosystems such as wetlands to maximise the provision of ecosystem services and enhance biodiversity [1, 23, 33]. Within the UK, for example, government policies and payment schemes to landowners encourage the creation, restoration and management of existing wetlands to increase biodiversity and foster local and regional flood resilience programs [22–24]. Water is a requirement for mosquito breeding, and thus there is an urgent need to understand how policy-driven changes in wetlands impact mosquito communities, as well as their interactions with animal and human hosts, and how this trades off with disease transmission risk [21, 38, 49, 51].

There is growing evidence globally that wetland management for biodiversity can affect mosquito communities [63], not only by changing aquatic breeding site characteristics and vegetation, but also via impacts on mosquito predators [37, 70], and that this can lead to public health co-benefits or dis-benefits depending on local context. Some studies have found that mosquito density increases after wetland construction and management [44, 72], but if implemented correctly, wetland management schemes that create diverse and permanent wetland habitats can decrease mosquito populations by simultaneously decreasing habitats suitable for larval mosquitoes while increasing those suitable for known mosquito predators [41, 65, 66].

Altering wetland water levels during the mosquito breeding seasons, including complete drying of water bodies, can lead to desiccation of mosquito larvae and prove an adequate control method, but these strategies can negatively impact other aquatic flora and fauna of wetlands [67]. In Australia, draining and re-filling of urban wetlands to manage an invasive fish species led to increased abundance of mosquito species compared with undrained urban wetlands [38]. In some contexts, integrated management for biodiversity and reduced public health risks and nuisance biting from mosquitoes has been possible. For example, integrated Marsh Management Schemes employed in salt marshes in the USA combine tidal flow restoration and vegetation management favouring fish and wildlife biodiversity with management of open water surfaces (open marsh water management) to enhance habitats for larvivorous fish predators [64].

In Europe and the UK, there is a dearth of data regarding the influence of wetland management on mosquito communities encompassing both nuisance biters and potential disease vectors [39]. There is some evidence that wetland creation can promote increased populations of various mosquito species, as demonstrated by studies on Aedes vexans in river flood plains [80] and on Aedes detritus in newly created salt marshes in England [16] However, existing research is limited in its examination of the potential trade-offs between conservation-oriented management practices aimed at preserving biodiversity and the subsequent implications for public, animal and wildlife health [49].

This knowledge gap is increasingly pressing for Europe, particularly considering the recent outbreaks of West Nile Virus (WNV). Between 2010 and 2018, there were more than 3500 reported human cases of West Nile fever in Europe, with infections distributed from Turkey to Spain and as far north as Germany, resulting in 379 deaths [82]. Furthermore, the heightened circulation of the Usutu virus across central western and central Europe, associated with mosquitoes in and around wetlands, adds urgency to the need for a better understanding of the impacts of wetland management on mosquito communities [26, 30].

Specifically, recent detection of Usutu virus in Southern England, impacting blackbird populations [31], combined with the proximity to ongoing West Nile virus transmission in Germany and the Netherlands [2], underscores an increased risk of further mosquito-borne pathogen incursions in the region. This risk is heightened by the high prevalence and overlap of the primary vector, *Culex pipiens* s.l., across Europe [55]. Studies conducted in UK fenlands have explored the links between wetland management and mosquito abundance and revealed that emergent vegetation and sediment build-up can lead to warmer waters and increased densities of Culicine mosquito species, while drainage of water levels can decrease Culicine abundance but create a more suitable habitat for *Anopheles maculipennis* s.l., a species complex known for its nuisance biting behaviour [52].

Combining empirical mosquito surveys with statistical spatial modelling of abiotic and biotic drivers of mosquito community composition across wetland management gradients may lead to a more detailed understanding of impacts of wetland management on candidate vector species and biting risks. Utilising such an approach in marshes in the east of England (North Kent Marshes), Golding et al. [35] found that ditch shrimp and fish predators reduced the prevalence of mosquito larvae, namely of *An. maculipennis* sensu lato (a species complex thought to include minor and historical malaria vectors) and *Culex modestus* (a bridge vector for WNV) and suggested that habitat management for these species could both increase biodiversity and reduce mosquito numbers.

Species distribution models have been applied at national, sub-national and local scales to study the impacts of wetland changes on individual mosquito vector species, but these ignore important species community interactions. However, community modelling approaches such as joint species distribution models [34, 60, 61] may offer great advantages. These models can help identify shared responses to environmental conditions [62] and account for potential biotic interactions such as competition and predation. Such interactions can strongly influence mosquito population dynamics and persistence [5, 8, 70] and will likely modulate individual vector species responses to wetland changes [63]. This study applies community joint modelling methods to sampled larval and adult mosquito population data in a large UK wetland that has been subject to management changes under agri-environmental schemes, where water levels, livestock grazing pressure and mechanical interventions are differentially managed, with the following objectives:

1. To understand the role of abiotic (physico-chemical water parameters, ditch morphology, vegetation structure) and biotic factors (predator communities) in determining larval mosquito community composition
2. To determine whether wetland management changes under recent agri-environmental schemes are likely to have increased the larval abundance and diversity of key UK mosquito vectors of important mosquito-borne viruses.

## Methods

### Study site

The Somerset Levels and Moors (SLM), the largest remaining lowland wet grassland in the UK, spanning 650 km^2^ in the south-west of England, holds unique ecological significance. Designated under the European Commission Habitats Directive and the UK Biodiversity Action Plan, it serves as an exemplary coastal grazing marsh habitat [45]. The SLM’s structure consists of interconnected water-filled ditches, locally known as rhynes. This coastal habitat, lying largely below or at sea level, forms a large catchment area for Somerset, and this matrix of rhynes drain land that would otherwise be too boggy for farming. The area plays a crucial role in providing essential ecosystem services to local communities and tourists, boasting high biodiversity with a notable presence of wading and migratory birds year round [1].

The SLM’s history is marked by periodic winter inundation over the past 10,000 years, contributing to the development of fertile peat soils and rich biodiversity. However, human activities, such as drainage and ditching for seasonal grazing pastures, began as early as the ninth century and intensified in the mid-twentieth century, reaching a peak with peat extraction and agricultural practices. Recognizing its environmental sensitivity, the SLM received designation as an Environmentally Sensitive Area (ESA) in 1987. Subsequently, agri-environmental schemes were implemented to support farmers in adopting management practices beneficial to biodiversity and flood management [58]. This led to the transformation of arable land back into wet grassland.

Operating within a tiered system, these agri-environment schemes prescribe different measures (Table 1). The entry-level option, tier 1, aims to preserve the plant and invertebrate communities in permanent grassland, which are sensitive to disturbance caused by ploughing and arable cropping. In contrast, tier 3, the most demanding management option, focusses on enhancing plant species diversity and habitat for breeding waders and overwintering wildfowl by promoting wet winter and spring conditions in permanent grassland. This tier encourages lower grazing pressure, minimizes mechanical intervention in fields and surrounding ditches and maintains higher minimum water levels, particularly during the winter months (tier 3 versus tier 1).

We anticipate that these tiered prescriptions will influence various ecological factors, such as shading, vegetation structure, ditch morphology and the presence of macro-invertebrate predators. Consequently, these conditions are expected to have a significant impact on mosquito species composition and abundance. Our study aims to sample and investigate the key drivers of mosquito community composition in tier 3 versus tier 1 sites across the Somerset Levels and Moors (SLM), shedding light on the ecological dynamics influenced by these contrasting wetland management practices. By comparing mosquito communities between the two tiers, we hope to gain valuable insights into how different management approaches affect mosquito populations and their associated ecological interactions.

**Table 1:** Overview of key differences in management prescriptions for tier 1 (permanent grassland) and tier 3 (raised water level areas)

Prescription category	Tier 1	Tier 3
Fertilizer application	Do not exceed existing level of inorganic fertilizer, and in any case, do not exceed 75 kg of nitrogen, 37.5 kg of phosphate and 37.5 kg of potash per hectare. Do not exceed existing level of home-produced organic fertilizer and do not apply any other organic fertilizer	Apply no inorganic fertilizer and do not exceed existing level of organic manure, provided it is only home-produced cattle farmyard manure and does not exceed 25 tonnes per hectare per annum. No slurry should be applied
Grazing	Graze with cattle or sheep but avoid poaching, under-grazing or over-grazing	Graze only with cattle but do not graze before 20 May in any year. Do not exceed a grazing density of one animal per 0.75 hectare from 20 May to 8 July. Do not cause poaching, over-grazing or under-grazing
Mowing and hay making	If you cut the grass for hay or silage, graze the aftermath	Do not make silage. Unless traditionally the land has been used just for grazing, each year mow at least one-third of the land (or mow 1 year in three) but not before 8 July. Do not graze the land prior to laying it up
Mechanical operations	You may use a chain harrow or roller, but no other form of cultivation is allowed. Maintain existing field gutters, surface piping, rig and furrow, ditches or rhynes by mechanical means, not sprays. Do not install additional surface piping	Mechanical operations prescribed in tier 1 are allowed, but not between 31 March and 1 July. Maintain existing field gutters, surface piping, rig and furrow, ditches or rhynes by mechanical means, not sprays. Do not install additional surface piping
Herbicide use	Do not use herbicides except to control specific weeds (creeping buttercup, soft rush, nettles, spear thistle, creeping or field thistle, curled dock, broad-leaved dock or ragwort). Apply herbicides by weed wiper or spot treatment	As in tier 1, but additionally do not use herbicides to control creeping buttercup
Water levels	Water levels in ditches and rhynes must be maintained at specified levels, with at least 15 cm of water in the bottom of the ditches/rhynes at all times	Water levels in ditches and rhynes must be maintained at not more than 30 cm below mean field level from 1 May to 30 November and at not less than mean field level from 1 December to 30 April to cause conditions of surface splashing

For a comprehensive overview of the management prescriptions, including specific guidelines and restrictions, please refer to Additional file 1 document ESA Tiered Rules

### Ecological survey

**Fig. 1:**
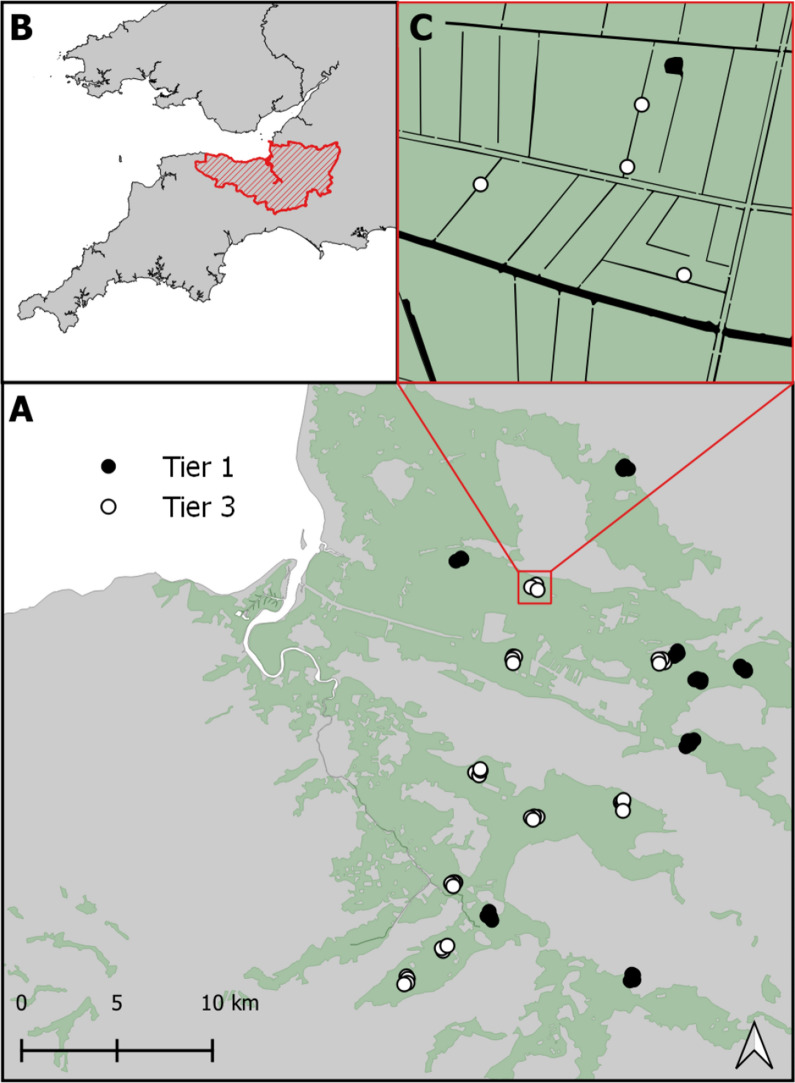
**A** Map of Somerset Levels and Moors study site. Extent of coastal grazing marsh in green with tier 1 and tier 3 locations with sampling sites superimposed (black and white circles, respectively). **B** Location of the study site (red hatching) in South England. **C** Inset frame showing detailed hierarchical spatial sampling design for each sampling site (circles) in which four ditches were sampled within a 500 m square radius for each location

We randomly selected 17 ditch locations across two management regimes, 8 in tier 1 management and 9 in tier 3 of the SLM (Fig. 1). At each site, we selected four sampling sites (ditches) within a 500 m radius of the location, often part of an interconnected ditch system. We surveyed these sampling sites using a standard dipping protocol across three timepoints: spring (May), summer (June/July) and autumn (August/September) for 3 years, from 2009 to 2011. We set up six dip-points, for which we took GPS locations at each sampling site along the ditch of 1–6 meters, randomly determined by the throw of a die. During each visit, we took a complete submersion dip sample from both water–body margins as well as the centre of the ditch using a 1-litre volume mosquito dipper at each of the dip-points.

We recorded the abundance of mosquito larvae and pupae, and that of potential mosquito predator groups at each dip -point. Aquatic macro-invertebrate species were identified in situ to order and suborder, where possible, using [25]. Mosquito larvae and pupae were preserved in 70% ethanol and identified to species or species complex level in the laboratory using the morphological keys by [4, 19, 73, 75]. During each visit, details of bankside, emergent and floating channel vegetation were recorded [18, 43]. Plants within and at the edges of the ditch were identified to genus or species level, and their percentage cover and height estimated. Vegetation height and percentage cover values were averaged across species in three groups on the basis of their functional impact, bank, emergent and floating vegetation, since these vegetation structures are likely to have differential impacts on habitat suitability across mosquito species (Table 2). We measured the physico-chemical characteristics of the ditch at each sampling site, assessing ditch width and area of the ditch shaded (a proxy for habitat openness) as well as pH, temperature, turbidity and salinity of the water. Average values for the covariates listed in Table 2 were summarised across the six dip-points per ditch site in each season.

**Table 2:** Effect of environmental variables on mosquito abundance: impact and expected ecological implications of key environmental factors on mosquito populations, including vegetation cover, water characteristics and habitat structure

Variable	Description	Impact on mosquito abundance
Floating vegetation cover (%)	The percentage of the water surface covered by floating plants or plants with significant leaf coverage	Expected negative impact on mosquito population density. Dense floating aquatic plants, such as *Lemna spp.*, can inhibit mosquito larvae and pupae reaching the water surface for air [20, 27]
Bank vegetation height (cm)	The height of vegetation along the ditch margins and banks	Increased structural complexity may provide favourable microhabitats for adult mosquitoes and increase availability of sheltered resting places [71]
Bank vegetation cover (%)	The percentage of banks covered by plant matter	Cover would be expected to provide similar benefits as height
Emergent vegetation height (cm)	The height of vegetation emerging vertically from the waterbody	Emergent vegetation may provide increased shelter for female mosquitoes ovipositing on the water surface and improve larval and pupal survival through predator avoidance [69]
Emergent vegetation cover (%)	The percentage area of emergent vegetation	Cover would be expected to provide similar benefits as height
Shaded water (%)	Percentage of water surface shaded	Expected to positively impact mosquito species with a preference for heavily vegetated or cool breeding sites. This characteristic also serves as an indicator of reduced habitat openness [39]
Width (cm)	Width of the waterbody	Wider waterbodies may be more favourable for vertebrate predators, negatively impacting mosquito density [76]. Some species, such as *An. maculipennis* s.l., prefer more open habitats [39]
Water temp (^∘^C)	Water temperature at sampling	Larval development time is shorter, and survival is better at moderately higher temperatures [3, 56, 68]
Dissolved O_2_ (ppm)	Concentration of dissolved oxygen in the water	Uncertain impact as many UK species are tolerant to a broad range of dissolved oxygen levels
pH	Water acidity/basicity at sampling	Most mosquitoes prefer neutral pH levels for optimum growth and are tolerant to moderate fluctuations [28]
Turbidity	Water clarity, indication of water flow	Turbid waters are expected to increase mosquito larvae survival since predator efficiency is reduced [12, 79]
Salinity (ppt)	Salt content of the water body	Salinity directly affects mosquito immature presence, with tolerance varying across species [54]

### Statistical analysis

We used a joint multivariate hierarchical generalized mixed linear model approach to account for the interdependency of species responses to the environment and species responses to each other in the ecosystem by modelling all species simultaneously and accounting for each species responses to measured and unmeasured environmental covariates through latent variable factors [81]. We fitted our model using the R package Hierarchical Modelling of Species Communities (HMSC; Ovaskainen et al. [60], Ovaskainen and Abrego [59]) framework, to explore how biotic and abiotic interactions drive mosquito larval distribution across the SLM.

The multi-species generalised linear latent variable model (with probit link function) was fitted to the presence–absence data for four mosquitoes and eight predator groups obtained from our 320 sampling sites with abiotic covariates on a linear scale (Table 2). We excluded any species that occurred fewer than ten times to increase statistical stability [59], leading to the exclusion of one mosquito species and four predator groups (see Results). To account for potential spatial biases in the sampling data, we generated a distance matrix, calculated from the average coordinates across the six dip-points that make up each sampling site, to represent the spatial scales between each sampling unit as a spatially structured random effect [29]. We considered the impact of temporal effects on sampling methods by including a nested random effect for both year and time point. In this case we consider the height and cover area of three different plant functional groups, bank, emergent and floating vegetation as an abiotic driver, as we expect them to function as a regulator of population fitness through shielding of predation or similar processes [69].

The model was fitted using four Markov chains Monte Carlo (MCMC) with a transient period of 5000 samples and target of 1000 samples per chain using a thinning rate of 1000 for a total of 4 million MCMC post-transient in samples in total. Parameter convergence was measured using Gelman and Rubens potential scale reduction factor (PSRF, Gelman and Rubin [32]). We used five-fold cross validation to validate model performance, comparing predictive and explanatory values of Tjur’s R2 and the area under curve (AUC) statistic for each species [48, 77]. We examined the importance of different sets of covariates in our model by partitioning the variation explained by fitting of partial models [7, 59]. We originally aimed to construct abundance models that included the same covariates, as we hypothesised that biotic interactions would have a greater impact on species abundance than species presence. However, due to the high complexity of the model, it was deemed computationally infeasible to achieve an acceptable fit, and run-times exceeded 1 month without reasonable convergence for all species [40].

To understand whether management tiers influence potential abiotic drivers of mosquito populations, we estimated the marginal effect of management tier on each covariate measured in our sampling procedure (Table 2). We modelled each covariate separately against management level as a categorical factor, with both a random effect for site and a nested random effect of season within year to account for temporal differences in covariate distribution. Bayesian multivariate models were built in the probabilistic programming language Stan using the BRMS package in R [11, 13]. Covariates measured on a percentage scale (metrics of vegetation cover and shaded area) used a zero-one inflated beta response distribution. Bank and emergent vegetation height used lognormal hurdle mixed response distributions to account for over-dispersion and the influence of zero values. All other covariates used a Student’s *t* distribution for robust estimation of parameter values. Significance was measured across the 95% CI using mean equal tailed intervals of the posterior distribution.

## Results

### Differences in environmental conditions between management tiers

Metrics of ditch vegetation structure differed significantly between sites subject to tier 1 versus tier 3 management, whilst physico-chemical properties of the waterbody and ditch structure parameters did not (Table 3). Though waterbodies were on average 8 cm wider in tier 3 managed areas, this difference was not statistically significant (95% CI [−23.94, 6.31]). There was no measurable difference in turbidity (95% CI [−0.15, 0.18]) or salinity (95% CI [− 0.12, 0.15]) between the management tiers, and pH values were on average −0.3 lower in tier 3 areas, but this was also non-significant (95% CI [0.03, 0.65]).

Bank vegetation was more likely to be present (95% CI [−0.29, −0.10]) (Additional file 2: Table S1), and when present it was significantly taller, by 25 cm on average (95% CI [−55.78, −4.24]), in tier 3 ditches than tier 1 ditches, but we found no differences in the levels of bank-side vegetation cover between tiers (mean = 0.01, 95% CI [−0.05, 0.07]). Similarly, we found that emergent channel vegetation was 29% more likely to be present in tier 3 areas (95% CI [−0.39, −0.18]), and when emergent vegetation was present it was 5 cm taller on average in tier 3 areas than in tier 1 areas (95% CI [−12.39, −0.34]). There was no measurable difference in the probability of floating vegetation cover being 0% (95% CI [−0.15, 0.06]) or 100% (95% CI [−0.1, 0.04]) between tiers, but on average there was 10% less floating vegetation cover in tier 3 areas than in tier 1 areas and this was significant (95% CI [0.01, 0.19]). The amount of shaded area of the channel did not vary significantly between tiers (mean = 0.12 95% CI [−0.01, 0.27]), but the probability of a waterbody being completely shaded was 48% higher in tier 3 than tier 1 areas (95% CI [−0.8, −0.2]), and the probability of a waterbody having no shade was 10% more likely in tier 3 areas (95% CI [0.03, 0.016]).

**Table 3:** Differences in environmental variables between the tier 1 (T1) and tier 3 (T3) wetland management regimes

Model covariate	Effect of tier 3	PD (%)	MPE	MPE_low_	MPE_High_
Salinity	–	57.40	0.01	−0.12	0.15
Emergent vegetation height (Height_Emerg_)	Taller emergent vegetation	98.41	−5.30	−12.39	−0.34
Dissolved oxygen (DO^2^)	–	89.17	6.59	−4.10	17.40
pH	–	96.53	0.30	−0.03	0.65
Turbidity	–	59.00	0.02	−0.15	0.18
Floating vegetation cover (Cover_Float_)	Less floating vegetation cover	98.33	0.09	0.01	0.19
Bankside vegetation cover (Cover_Bank_)	–	76.62	0.01	−0.05	0.07
Emergent vegetation cover (Cover_Emerg_)	–	70.88	−0.01	−0.04	0.02
Water temperature	–	88.33	−0.82	−2.24	0.59
Shaded	–	96.37	0.12	−0.01	0.27
Ditch width	–	87.67	−8.58	−23.94	6.31
Bank vegetation height (Height_Bank_)	Taller bank vegetation	99.62	−25.28	−55.78	−4.24

The table presents the marginal effect of management tier for each environmental covariate from pairwise posterior distribution contrasts of T1–T3 values. Probability of direction (PD) estimates above 97.5 are deemed significant and highlighted in bold. Mean parameter estimates (MPE) with lower MPE_Low_ and upper MPE_High_ estimates represent the equal tailed 95% CI estimate across the model’s posterior distribution. Full parameter estimates for each model covariate are given in Additional file 2: Table S1

### Abundance and prevalence of sample mosquito and predator taxa

We recorded 12 different aquatic macro-invertebrates taxa in the SLM, of which 5 were mosquitoes (Table 4). We identified 6896 mosquito larvae in total. *Culiseta annulata* (*n* = 3250, 47.13%) and *Culex pipiens* (*n* = 3248, 47.10%) made up the highest proportion of these larvae, followed by *Anopheles claviger* (*n* = 292, 4.23%) and *Anopheles maculipennis* s.l. (*n* = 105, 1.52%). *Anopheles maculipennis* s.l. were most prevalent, occurring in 13% of the sample sites, followed by *Cs. annulata* (12%), *An. claviger* (11%), *Cx. pipiens* (10%) and lastly *Aedes (Ochlerotatus) caspius*, which was present in just a single sampling site (< 0.1%). Because of the low abundance and low prevalence, *Ae. caspius* was omitted from the subsequent analysis.

We identified eight potential predator taxa that were present in at least ten sites to be included in this statistical analysis (Table 4). Of these taxa, adult Coleoptera (water beetles) were most prevalent, being present in the most sampling units (27%, *n* = 308 predator individuals recorded), followed by Corixidae (water boatmen), which were also the most abundant predator species (26%, *n* = 647), Zygoptera larvae (damselflies, 25%, *n* = 349) and Coleoptera larvae (19%, *n* = 139). The other four taxa had a much lower prevalence and abundance across all sampling units, including Gammaridae (ditch shrimp, 8%, *n* = 103), Anisoptera larvae (dragonflies, 5%, *n* = 31), *Ilyocoris cimicoides* (saucer bugs, 3%, *n* = 19) and *Nepa cinerea* (water scorpions, 3%, *n* = 11).

**Table 4:** Relative prevalence (rate of occurrence across all sites) and total (and proportional) abundance of mosquito and predator taxa across sampled sites among sampled individuals across study sites

Taxon	Prevalence (%)	Abundance (total)	Mean abundance per sample site
*Anopheles maculipennis* s.l.	13	105	2.44 ± 2.22
*Anopheles claviger*	11	292	8.11 ± 11.84
*Culex pipiens* s.l.	10	3248	101.50 ± 244.43
*Culiseta annulata*	13	3250	81.25 ± 160.62
Corixidae	26	647	7.70 ± 20.89
Coleoptera larvae	19	139	2.24 ± 1.70
Coleoptera adults	27	308	3.58 ± 3.30
Zygoptera larvae	26	349	4.20 ± 5.55
Anisoptera larvae	5	31	1.82 ± 1.42
*Ilyocoris cimicoides*	3	19	1.90 ± 2.18
*Nepa cinerea*	3	11	1.10 ± 0.32
Gammaridae	8	103	4.29 ± 4.65

### Overall accuracy of community models and partitioning of variance between key sets of drivers

Parameter convergence of the HMSC model was satisfactory, with all chains generating sufficient effective samples and PSRF values (Additional file 2: Fig S1 ). Explanatory AUC values (for the training dataset) were high for all mosquito species (0.86−0.99) and predictive AUC values (from the cross-validation) were reasonable (0.75–0.89). Explanatory AUC values were similarly high for potential predator taxa, but predictive AUC values were much lower for some of the less abundant taxa (*Nepa cinerea* = 0.4, *Ilyocoris cimicoides* = 0.55, *Anisoptera* larvae = 0.55, *Coleoptera* larvae = 0.57). All other predator taxa had adequate predictive AUC values above 0.69 (Table 5).

**Fig. 2:**
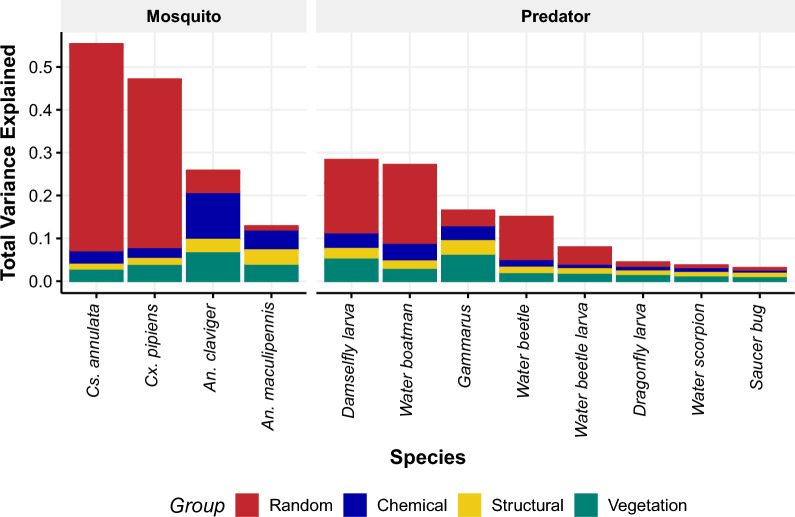
Variance partitioning and total variance explained by each component for larval mosquitoes and predator species prevalent in the study side. Random effects are the variance explained by year season and the spatial component of the model summed for each species. The chemical category includes the physico-chemical covariates pH, dissolved oxygen, salinity, turbidity and water temperature; the structural category includes the width and relative shadiness of the water body; and the vegetation category includes all vegetation metrics—floating, emergent and bank—as covariates in the model. Detailed breakdown of the variance explained by the random effects is provided in Additional file 2: Table S2

Metrics of variance explained for the training dataset were higher for Culicine species (*Cx. pipiens *s.l. Tjur’s R^2^ = 0.47; *Cs*. *annulata* Tjur’s R^2^ = 0.55) than Anopheline species (*An*. *maculipennis* s.l. Tjur’s R^2^ = 0.12; *An*. *claviger* (Tjur’s R^2^ = 0.23). When examining the importance of different sets of covariates, for mosquito species, we found that spatiotemporal effects accounted for on average 43% (SD 29%) of all variation explained by the models (Fig 2, Additional file 2: Table S2 ). Tjur’s R^2^ values for predator taxa were much lower than for the mosquito species, except for Corixidae (Tjur’s R^2^ = 0.27) and Zygoptera (Tjur’s R^2^ = 0.28) larvae.

Random effects accounted for substantial variation in Culicine species and low amounts of variation for Anopheline species (Fig. 2). For the Anopheline species, a higher proportion of variance was explained by chemical and channel structure covariates than for Culicine species. Temporal effects of year and season explained less variation in presence of mosquito species compared with the predator taxa, and little in Anopheline species (Additional file 2: Table S2)

**Table 5:** Accuracy with which community models explained and predicted the distributions of mosquito and predator taxa including area under curve (AUC) and Tjur’s R^2^ values for explanation and prediction

Taxa	Explanatory		Predictive	
	AUC	R^2^	AUC	R^2^
*Anopheles maculipennis* s.l.	0.86	0.12	0.75	0.06
*Anopheles claviger*	0.91	0.23	0.84	0.14
*Culex pipiens* s.l.	0.98	0.40	0.84	0.22
*Culiseta annulata*	0.99	0.53	0.89	0.38
Corixidae	0.88	0.27	0.82	0.21
Coleoptera larvae	0.78	0.08	0.57	0.02
Coleoptera	0.81	0.15	0.69	0.08
Zygoptera larvae	0.89	0.27	0.80	0.19
Anisoptera larvae	0.86	0.03	0.55	0.00
*Ilyocoris cimicoides*	0.87	0.03	0.50	0.00
*Nepa cinerea*	0.99	0.04	0.40	−0.01
Gammaridae	0.87	0.14	0.76	0.08

### Larval mosquito responses to environmental drivers

**Fig. 3:**
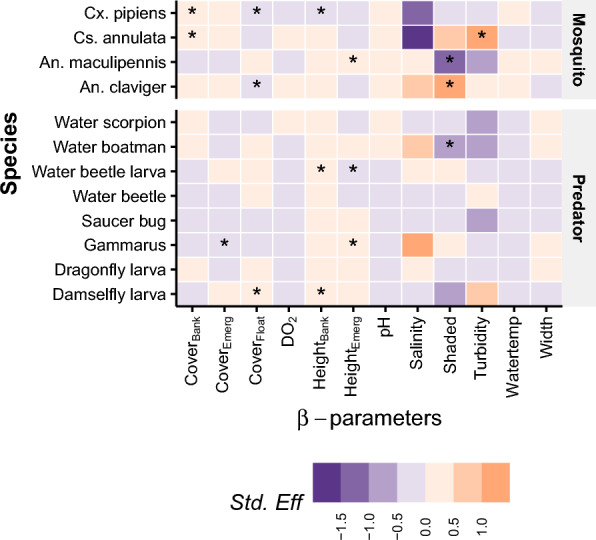
Species responses to covariates by standardised coefficient effect size (SE). *Indicates significance of parameter and cells that are blank indicate where the effect was not significant, that is, the 90% credible interval bridged zero. Height_Emerg_ = Emergent Vegetation height, Height_Bank_ = Emergent Vegetation height, Cover*Float* = Floating Vegetation cover, Cover_Bank_ = Bank Vegetation height, Cover_Emerg_ = Emergent Vegetation cover

*Culex pipiens* was significantly positively associated with bank vegetation cover (mean = 0.03, 90% CI [0.01, 0.04]), negatively associated with bank vegetation height (mean = −0.006, 90% CI [−0.017, −0.001]), and negatively associated with floating vegetation cover (mean = −0.01 90% CI [−0.018, −0.001]) (Fig. 3). *Culiseta annulata* was significantly positively associated with more bankside vegetation cover (mean = 0.02, 90% CI [0.001, 0.035]) and high turbidity areas (mean = 1.41, 90% CI [0.28, 2.65]). *Anopheles maculipennis* s.l. showed strong preference for habitats with little shade (mean = −1.13, 90% CI [−2.20, −0.13]) and higher levels of emergent vegetation (mean = 0.015 90% CI [0.004, 0.026]) (Fig. 3).* Anopheles claviger* exhibited a strong preference for shaded habitats (mean = 1.23, 90% CI [0.34, 2.17]), and ditches with little floating vegetation cover (mean = −0.015 90% CI [−0.025, −0.006]) (Fig. 3).

Several potential predator taxa were also significantly correlated with an array of physico-chemical and vegetation drivers (Fig. 3), but we only interpret these further for those predatory taxa for which a larger percentage of variance in occurrence was explained by the model, namely water boatmen and damselfly larvae, Fig. 2). The probability of occurrence of water boatmen was significantly negatively associated with lower shading of water bodies (mean = −0.96, 90% CI [−1,74, −0.23]). The probability of occurrence of damsel fly larvae was significantly positively impacted by higher levels of floating (mean = 0.009, 90% CI [0.003, 0.015]) and height of bank vegetation (mean = 0.005, 90% CI [0.001, 0.009], Fig. 3).

### Residual association between species

**Fig. 4:**
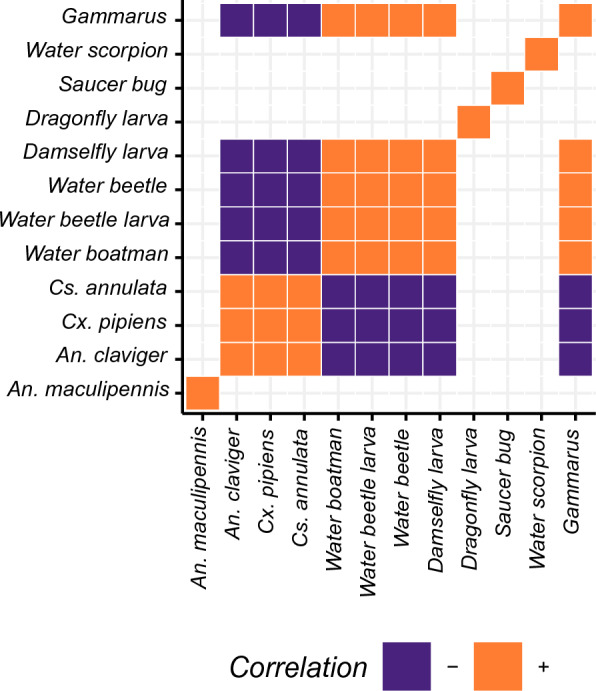
Significant species residual correlations drawn from the $\Omega$ model parameter for each species in HMSC. Coloured regions show which species meet the 95% significance threshold and are expected to be found together more than can be explained by model covariates alone. Correlations that do not meet this significance threshold are blank

We found significant positive residual species associations between all mosquito species except *An. maculipennis* s.l. after accounting for environmental responses in the HMSC community model (Fig. 4). Additionally, we found that all species of mosquito except *An. maculipennis* s.l. show significant negative associations with potential predator taxa including water beetle larvae and adults, and damselfly larvae, water boatmen and *Gammarus* spp. Saucer bugs, dragonfly larvae and water scorpions do not show any significant associations with any other species. All other predator taxa show significant positive associations with one another (Fig. 4).

## Discussion

### Vegetation structure as a key driver of mosquito communities including potential vectors

Increased water levels in tier 3 areas have been previously shown to favour the establishment of wetland meadow plant species, which increase the diversity and quality of vegetation in these areas compared with tier 1 areas [1]. Our study supports this, with tier 3 areas leading to significant increases in emergent and bankside vegetation height, increasing the structural complexity of vegetation compared with tier 1 areas (Table 3).

Areas such as wetlands and marshes tend to harbour a wide variety of mosquito species, due to the presence of a variety of suitable water bodies for oviposition, and aquatic plants that provide shelter, food and protection from predators, as well as a diverse set of host species from which to draw blood meals [4, 53]. Adult mosquitoes benefit from vegetation that is structurally complex, consisting of plant species communities that create shaded and sheltered micro-habitats that protect the mosquitoes from direct sunlight, wind and other environmental stressors. Such conditions enhance overall habitat suitability for adult mosquitoes [4]. Juvenile mosquitoes may also perceive similar benefits from the underwater structures of algae and plant roots as refuges from predators [17]. A review by Rey et al. [63] found that wetlands with high vegetative complexity had a greater diversity of mosquito species compared with wetlands with low vegetative complexity. Consistent with these prior studies, we found that occurrence of three of the key mosquito species in the study area (*An. claviger* and *An. maculipennis* s.l., and to a lesser extent, the *Cx. pipiens* complex) was favoured by more complex ditch vegetation structure characteristic of tier 3 management (increased height and cover of emergent and bankside vegetation, Table 3). Consistent with the associations described by Hawkes et al. [39], *An. maculipennis* s.l. showed significant preference for less shaded environments, suggesting a preference for open style habitats, while *An. claviger* showed a preference for heavily shaded habitats (Fig. 3). For *Cx. pipiens* and*An. claviger*, both of which can cause significant biting nuisances, tier 3 areas are likely to offer more favourable conditions because of these species’ preference for little floating vegetation cover (Fig. 3, Table 3). Floating vegetation can provide a physical barrier between mosquito and oviposition site as well as larvae and air, dissuading oviposition in these areas [27]. Yet, previous studies have found positive associations between floating vegetation cover and mosquito species presence, suggesting the impacts of this factor on mosquito larvae is complex and context dependent [20, 35].

Except for the association of turbid water with *Cs. annulata* presence, no significant effects of physico-chemical characteristics of the water on mosquito occurrence were found (Fig. 3). This aligns with prior knowledge that Culicine species, *Cx. pipiens* and *Cs. annulata* utilise a breadth of oviposition sites, including drainage ditches, artificial containers and small stagnant waters, which vary widely in water parameters [39]. We found that physico-chemical factors had a larger contribution to variance explained for the Anopheline species *Anopheles maculipennis* s.l. and *An. claviger*, at 11% and 6%, respectively, suggesting more restricted oviposition site preferences. The SLM system is an interconnected network of ditches that covers an area over several hundred square kilometres, leading to relatively homogeneous water chemistry across our study area. This means that the range of conditions experienced by our sampled species might not be large enough to elucidate any meaningful differences in water parameter preferences (and indeed the Tier management regimes did not differ significantly in physico-chemical conditions).

### Biotic drivers of larval mosquitoes

Consistent with prior studies of mosquito community composition at landscape level, we found that biotic interactions may affect the distribution of mosquitoes across a wetland environment [35]. Many of the potential predator taxa such as dragonfly and damselfly larvae are frequently observed as effective larval mosquito predators in other contexts, and indeed, some such as dragonfly larvae have been investigated for biological control of mosquitoes [50, 70] (Onyeka 1983). Water beetles and water boatmen have also been implicated in mosquito larval predation, but their relative predation pressure is thought to be linked to the vulnerability of mosquito larvae [42, 50].

As described above, vegetation structure in and around waterbodies affects the availability of refugia from predators, and consequently the effectiveness of predator avoidance strategies of immature mosquitoes [69]. Environments with complex underwater vegetation limit the space for predators and mosquito larvae to interact and reduces overall predator efficiency [69, 76]. The higher cover and height of emergent vegetation detected in tier 3 areas could provide complex vegetation structure both above and below the water level, providing shady refugia that improve predator avoidance in these sites.

It is crucial to recognise that the species interactions deduced from residual correlations in joint occurrence models are not as dependable as direct observations of predator–prey interactions. Instead, these inferred interactions may be indicative of unmeasured factors such as shared or non-shared environmental preferences between species [62]. In essence, while joint occurrence models provide valuable insights, caution should be exercised in attributing the correlations solely to direct predator–prey interactions, as other environmental factors might contribute to the observed patterns [83]. For example, though some mosquito species were found to be negatively correlated with *Gammarus* species, we suspect this may reflect different preferences for unmeasured environmental conditions. *Gammarus pulex* and other *Gammarus* species are omnivorous and occupy different depths of the waterbodies compared with mosquito larvae, leading to limited potential predation opportunities.

The community models exhibited relatively low performance for predator species compared with mosquito species. Therefore, to comprehensively grasp how wetland management may influence predator effects on mosquito populations in this context, additional and more detailed data on predators, with improved taxonomic resolution, could be valuable. Prior studies seem to suggest that management plans targeting biodiversity, such as tier 3, have been suggested to positively impact the abundance of key predator taxa, including fish [14, 37]. Increased predator abundance would provide a potential control agent for mosquito populations, but few studies have shown this in the field, and none in the UK [37, 50, 70]. Our study indicates that water beetle larvae and adults, dragonfly and damselfly nymphs and water boatmen may be key predator taxa that play a role in regulating mosquito populations within lowland wet grasslands, and that these roles should be investigated further to fully understand trade-offs between biodiversity management and mosquito biting risk.

## Conclusions

We have shown here how management schemes directed at increasing the biodiversity of grazed wetlands could increase the suitability of those habitats for the immature of some key mosquito vectors and nuisance biters, encouraging diverse vegetation structure in and around water bodies, which may reduce their vulnerability to predators. However, thinning or removal of vegetation is not a viable strategy to control mosquito populations, as it is at odds with the targets of wetland management strategies. Vegetation removal impinges upon important wetland ecosystem functions by decreasing biodiversity, lowering water quality and reducing flood resilience of an area [1, 64].

Furthermore, to interpret disease risk given future incursions of viruses such as West Nile virus, Sindbis virus or Usutu virus into the UK, it would be necessary to understand how these impacts of wetland management on juvenile mosquito populations cascade through into impacts on the ratio of adult vectors to susceptible hosts (a key parameter in disease transmission [74]), by sampling adult vectors, hosts and their interactions (e.g. via blood meal analysis) across wetland gradients into areas of human habitation [38]. This would provide the evidence-base for co-development of integrated mosquito management and risk awareness strategies among cross-sectoral stakeholders, which would minimise risk of exposure while aligning with environmental wetland management goals [49]. Given the diverse and growing mosquito-borne pathogen threats to people living in and around wetland ecosystems, and the diverse assemblages of potential mosquito vector species involved, the combination of joint models with empirical surveys provides an effective way of inferring the complex ecological interactions that will underpin the trade-offs between disease risk and wetland management.

### Supplementary Information

**Additional file 1.** Management tier prescription.+

**Additional file 2: Fig. S1.** Fitted model convergence metrics for the Beta, Omega and Gamma parameters of the HMSC model. Effective sample size (ESS) over 1000 indicate good fit, while potential scale reduction factor (PSRF) values of under 1.1 (though ideally 1.01) are considered converged for MCMC sampling. **Table S2.** Detailed variance partitioning results for taxa across different environmental components, including Chemical, Vegetation, Structural, and Random Effects (spatial, season, year). Values represent the proportion of variance explained by each component for the respective taxa.

## Data Availability

Data and reproducible code are available on GitHub (https://github.com/dansmi-hub/Smith2023/tree/master).
