# Supplementary material for: Vegetation structure drives mosquito community composition in UK’s largest managed lowland wetland
Source: Parasit Vectors. 2024 May 6;17:201. doi: 10.1186/s13071-024-06280-y (PMC11071336; doi:10.1186/s13071-024-06280-y)
Supplement: Supplementary file 1 — Additional file 1. Management tier prescription.+ [file 13071_2024_6280_MOESM1_ESM.pdf]

ECIF

Background to the ESA

1. The ESA extends over 29,260 hectares of the central Somerset lowlands, bounded by the Mendips to the north, low limestone escarpments to the east, the Blackdown Hills to the south and the Quantock Hills to the west.The moors comprise an extensive area of very low-lying basin peat, with a few remnants of raised bog, surrounded by alluvial clay and silt. The peat is overlain by riverine clay. Westwards from the moors lies an extensive area of slightly higher estuarine alluvium known as the Levels, most of which is excluded from the ESA. Grassland predominates and, traditionally, has been used for summer cattle grazing and hay cutting.

2. The whole area forms the largest remaining lowland wet grassland system in Britain and is consequently of outstanding environmental interest. The ecological interest is associated with the wet, often species-rich pastures and meadows and the surrounding network of ditches with their aquatic flora and invertebrate interest. This wet grassland area supports overwintering wildfowl and breeding waders for which part of the area is designated as a Ramsar/SPA site. The landscape value lies in the rectilinear pattern of traditionally managed fields and drainage channels within a low-lying wet and expansive grassland area. In addition, there is a wealth of archaeological interest, ranging from prehistoric wooden trackways to more recent buildings and structures.

3. In the 1970s and early 1980s the drainage of large areas of the Moors was improved. This, along with the increased use of chemicals and fertilisers resulted in the grassland being improved or converted to arable. This threat has been countered by the designation of 13 moors as Sites of Special Scientific Interest (SSSIs) and the establishment of the area as an ESA.

4. This is a 'part-farm' scheme which started in 1987. In 1992 it was extended by 530 ha and by a further 1,580 ha when the revised scheme was introduced in 1997. Overall uptake at the end of 1998 was c. 16,748 hectares.

5. A priority objective is to sensitively manage the grassland and water levels in the surrounding ditches (most tiers). Tier 1a helps to achieve enhancement by protecting the semi-improved, improved and unimproved species rich grassland through reduced inputs. To help achieve enhancement a new tier (Tier 1A) has been introduced to protect the semi-improved and unimproved species-rich grassland, through reduced inputs. The management requirements are similar to Tier 2, but without the water level restrictions. A water level supplement is also available on a site-specific basis, and is designed to benefit birds or rare plant species. These tiers and supplements are the main mechanism for achieving the Biodiversity Action Plan (BAP) targets in the area.

6. A further objective is to maintain the traditional landscape character, including the field boundary and historic features, by encouraging appropriate grassland management (all tiers). Enhancement of features such as ditches, pollarded willows, gates/wing fences and historic artefacts is encouraged through the Conservation Plans.

7. The all-year penning supplement (for Tiers 1, 1A & 2) has been introduced to maintains summer penning levels throughout the winter to protect the peat resource and its associated archaeological remains.

8. A buffer strip supplement has been introduced for arable land to creates fertiliser-free grass buffer strips adjacent to water courses. These are designed to reduce the run-off of agricultural inputs into the ditches, thus protecting the diverse, aquatic plant and insect communities.

Tier 1 Permanent grassland

Scheme Prescriptions

1. Maintain grassland, do not plough, level or reseed land. You may use a chain harrow or roller but no other form of cultivation is allowed.
2. Graze with cattle or sheep but avoid poaching, under-grazing or over-grazing.
3. If you cut the grass for hay or silage, graze the aftermath.
4. Do not exceed your existing level of inorganic fertiliser and in any case do not exceed 75kg of nitrogen, 37.5kg of phosphate and 37.5kg of potash per hectare (60 units of nitrogen, 30 units of phosphate and 30 units of potash per acre). Do not exceed your existing level of home produced organic fertiliser and do not apply any other organic fertiliser.
5. Do not use fungicides or insecticides.
6. Do not apply herbicides except to control creeping buttercup, soft rush, nettles, spear thistle, creeping or field thistle, curled dock, broad-leaved dock or ragwort. Apply herbicides by weed wiper or spot treatment
7. Do not apply lime, slag or any other acidity reducing substance.
8. Do not install under-drainage, do not mole drain, and do not subsoil or tunnel plough. Do not substantially modify your existing drainage system.
9. Maintain existing field gutters, surface piping, rig and furrow, ditches or rhynes by mechanical means, not sprays. Do not install additional surface piping.
10. Do not spray irrigate your land.
11. Maintain hedges, trees and pollarded willows in accordance with local custom.
12. Do not plant any additional trees or allow natural establishment of additional trees/ bush without prior agreement.
13. Do not damage or destroy any features of historic interest.
14. Obtain written advice on siting and materials before constructing buildings, roads or any other engineering operations which do not require planning permission or prior notification determination by the Local Planning Authority.
15. Maintain existing gates with wing fencing but do not erect any additional permanent fencing without prior consent.
16. Water levels in ditches and rhynes must either be:

- From 1 April to 31 October maintained at or above the penning level, provided since 1987, by the relevant Internal Drainage Board (IDB) or the Environment agency (EA) (as appropriate) and from 1 November to 31 March maintained at or above the winter level provided since 1987 by relevant IDB or the EA (as appropriate) with at least 15 cm 15 cm (6") of water in the bottom of the ditches/rhynes at all times.

Or, to obtain a supplementary payment:

- From 1 May to 30 November water levels in ditches and rhynes must be maintained at not more than 30 cm (12") below mean field level and from 1 December to 30 April maintained at not less than mean field levels so as to cause conditions of surface splashing.

17. Agreement holders must not pump below these levels which will be fixed for reference to gauge boards set to Ordnance Datum Newlyn.

18. You must abide by the Codes of Good Agricultural Practice (Annex IVII) for the Protection of Water, Soil and Air, published by the Ministry (references PB 0587, PB 0617 and PB 0618) as amended from time to time.

Agronomic Impact
1. No significant consequences for Income Forgone.
2. No significant consequences for Income Forgone.
3. No significant consequences for Income Forgone.
4. As a consequence of not being able to reseed and a required reduction in fertiliser application from the pre-ESA rate of (200 kg N, 40 kg P_2O_5, 30 kg K_2O)/ha to (75 kg N, 37½ kg P_2O_5, 37½ kg K_2O)/ha, the stocking rate will be reduced from 1.65 GLU/ha to 1.2 GLU/ha. This results in a decrease in livestock gross margin. Costs will be saved from reduced forage inputs, labour and interest on working capital.
5. No significant consequences for Income Forgone.
6. Typically this will involve a switch from using cheaper hormone based herbicides on an overall basis to spot treatment or wick application of more expensive chemicals. Both spot and wick application methods are more labour intensive techniques. Topping is also used to control weeds in both the non-ESA and ESA situation.
7. No significant consequences for Income Forgone.
8. No significant consequences for Income Forgone.
9. ESA agreement holders will have to clean out their ditches and rhynes at a greater frequency than non-agreement holders.
10. No significant consequences for Income Forgone.
11. Extra costs of hedge management involving hedge laying on a 15 year cycle and regular hedge trimming traditional to the area. Additional costs will be incurred for maintenance of pollarded willows in accordance with the local custom. Little pollarding is undertaken by non-ESA farmers.
12. No significant consequences for Income Forgone.
13. No significant consequences for Income Forgone.
14. No significant consequences for Income Forgone.
15. Extra costs associated with the maintenance of wooden gates and winged fencing compared to replacement steel gates.
16. No significant consequences for Income Forgone.
17. No significant consequences for Income Forgone.
18. No significant consequences for Income Forgone

Tier 1A - Extensive permanent grassland

Scheme Prescriptions

Observe prescriptions 1-18 plus additional prescriptions set out below:
19. Do not use a chain harrow or roller between 31 March and 1 July.
20. Do not exceed your existing level of inorganic fertiliser and in any case do not exceed 25kg of nitrogen, 12.5kg of phosphate and 12.5kg of potash per hectare (20 units of nitrogen, 10 units of phosphate and 10 units of potash per acre) each year.
21. Unless traditionally the land has been used for grazing each year mow at least one third (or one year in three) of the land but not before 1 July and do not graze the land prior to laying it up.
22. Do not cut or top the grass after 31 August.
23. Do not graze with sheep from 1 September to 1 March.
24. Do not use herbicides to control creeping buttercup.
25. Water levels in ditches and rhynes must be:

- From 1 April to 31 October at or above the penning level, provided since 1987, by the relevant IDB or the EA (as appropriate) and from 1 November to 31 March maintained at or above the winter level provided since 1987 by relevant IDB or the EA (as appropriate) with at least 15 cm (6") of water in the bottom of the ditches/rhynes at all times.

26. Agreement holders must not pump below these levels which will be fixed by reference to gauge boards set to Ordnance Datum Newlyn.

Agronomic Impact

19. 20A small amount of sward deterioration will result in a slight reduction in stocking rate. This coupled with a reduction in the quantity of fertiliser applied from (200 kg N: 40 kg P_2O_5 :30 K_2O)/ha to (25 kg N: 12 ½ kg P_2O_5, 12 ½ kg K_2O)/ha will decrease the stock carrying capacity. This in conjunction with the maintenance of higher water levels will reduce the stocking rate from 1.65 GLU/ha to 1.0 GLU/ha. Reduced forage inputs, requirement for labour and interest on working capital will produce cost savings.
20. As for prescription 19.
21. This will result in potential loss of land for silage production. The hay produced will be of lower energy value than silage and therefore extra concentrate feed will be required.
22. No significant consequences for Income Forgone.
23. This will result in loss of winter grazing for sheep and therefore incur extra costs associated with winter keep.
24. No significant consequences for Income Forgone.
25. No significant consequences for Income Forgone.
26. No significant consequences for Income Forgone

Tier 2 - Wet permanent grassland
Scheme Prescriptions

Observe prescriptions 1-18 plus additional prescriptions set out below:

27. Do not use a chain harrow or roller between 31 March and 1 July.
28. Do not exceed your existing level of inorganic fertiliser and in any case do not exceed 25kg of nitrogen, 12.5kg of phosphate and 12.5kg of potash per hectare (20 units of nitrogen, 10 units of phosphate and 10 units of potash per acre) each year.
29. Unless traditionally the land has been used just for grazing each year mow at least one third (or one year in three) of the land but not before 1 July and do not graze the land prior to laying it up.
30. Do not cut or top the grass after 31 August.
31. Do not graze with sheep from 1 September to 1 March.
32. Do not use herbicides to control creeping buttercup.
33. Water levels in ditches and rhynes must be either:

From 1 April to 31 October maintained at or above the penning level, provided since 1987 by the relevant IDB or the EA (as appropriate) and in any case not more than 45 cm (18") below mean field level and from 1 November to 31 March, maintained at or above the winter level provided since 1987 by the relevant IDB or the EA (as appropriate) with at least 30 cm (12") of water in the bottom of the ditches/rhynes at all times.

Or, to obtain a supplementary payment:

From 1 May to 30 November water levels in ditches and rhynes must be maintained at not more than 30 cm (12") below mean field level and from 1 December to 30 April, maintained at not less than mean field level so as to cause conditions of surface splashing.

34. Agreement holders must not pump below these levels which will be fixed by reference to gauge boards set to Ordnance Datum Newlyn.

Agronomic Impact

27.A small amount of sward deterioration will result in a slight reduction in stocking rate. This coupled with a reduction in the quantity of fertiliser applied from (200 kg N :40 kg P_2O_5 :30 K_2O)/ha to (25 kg N: 12 ½ kg P_2O_5 , 12 ½ kg K_2O)/ha will decrease the stock carrying capacity from 1.65 GLU/ha to 0.9 GLU/ha and therefore produce a substantial decrease in the livestock gross margin. Reduced forage inputs, the requirement for labour and interest on working capital will produce cost savings.
28. As for prescription 27.
29. Mow one third of land each year. This will result in potential loss of land for silage production. The hay produced will be of lower energy value than silage and therefore extra concentrate feed will be required.
30. No significant consequences for Income Forgone.
31. Do not graze with sheep from 1 September to 1 March. This will result in loss of winter grazing for sheep and therefore incur extra costs associated with winter keep.
32. No significant consequences for Income Forgone.
33. No significant consequences for Income Forgone.
34. No significant consequences for Income Forgone

Tier 3 - Permanent grassland raised water level areas
Scheme Prescriptions

Observe prescriptions 1-18 plus additional prescriptions set out below:
35. Do not carry out mechanical operations between 31 March and 1 July.
36. Apply no inorganic fertiliser and do not exceed your existing level of organic manure provided it is only home-produced cattle farmyard manure and does not exceed 25 tonnes per hectare (10 tones per acre) per annum. No slurry should be applied.
37. Graze only with cattle but do not graze before 20 May in any year.
38. Do not exceed a grazing density of one animal per 0.75 hectare (one animal per 1.8 acres) from 20 May to 8 July. Do not cause poaching, over-grazing or under-grazing.
39. Do not make silage. Unless traditionally the land has been used just for grazing each year mow at least one third of the land (or mow one year in three) but not before 8 July. Do not graze the land prior to laying it up.
40. Do not cut or top grass after 31 August.
41. Do not use herbicides to control creeping buttercup.
42. Water levels in ditches and rhynes must:
From 1 May to 30 November be maintained at not more than 30 cm (12") below mean field level and from 1 December to 30 April, maintained at not less than mean field level to cause conditions of surface splashing.
43. To further the objective of conserving, enhancing or protecting landscape, wildlife and historical features the Minister may specify different water level requirements.
44. Agreement holders must not pump below these levels which will be fixed by reference to gauge boards set to Ordnance Datum Newlyn.

Agronomic Impact

35. A small amount of sward deterioration will result in a slight reduction in stocking rate and the prohibition of inorganic and organic fertiliser will only permit a very low stocking rate of 0.25 GLU/ha. This will result in an 80% reduction of livestock gross margin. Reduced forage inputs, labour and interest on working capital will produce cost savings.

36. As for prescription 35.
37. No significant consequences for Income Forgone.
38. No significant consequences for Income Forgone.
39. Hay can only be made after 8 July. This will result in a reduction in forage digestibility. As a result, extra feed will have to be purchased as feed barley.
40. No significant consequences for Income Forgone.
41. No significant consequences for Income Forgone.
42. Surface splash conditions must be maintained from 1 December to 30 April. This will result in a loss of winter grazing, and therefore incur extra costs associated with winter keeping. Even after drainage surface conditions remain too wet to permit grazing before 20 May. In some years grazing cannot be carried out until early June. This will result in the loss of early spring grazing and restrict the growing season. This prescription will result in the replacement of agricultural grasses with less productive native species.
43. No significant consequences for Income Forgone.
44. No significant consequences for Income Forgone
