# Supplementary material for: Vegetation structure drives mosquito community composition in UK’s largest managed lowland wetland
Source: Parasit Vectors. 2024 May 6;17:201. doi: 10.1186/s13071-024-06280-y (PMC11071336; doi:10.1186/s13071-024-06280-y)
Supplement: Supplementary file 2 — Additional file 2: Fig. S1. Fitted model convergence metrics for the Beta, Omega and Gamma parameters of the HMSC model. Effective sample size (ESS) over 1000 indicate good fit, while potential scale reduction factor (PSRF) values of under 1.1 (though ideally 1.01) are considered converged for MCMC sampling. Table S2. Detailed variance partitioning results for taxa across different environmental components, including Chemical, Vegetation, Structural, and Random Effects (spatial, season, year). Values represent the proportion of variance explained by each component for the respective taxa. [file 13071_2024_6280_MOESM2_ESM.pdf]

SI: Vegetation structure drives mosquito community composition in UK's largest managed lowland wetland

Table of contents

Figure S1 2
Table S2 3

Figure S1

Fitted model convergence metrics for the Beta, Omega and Gamma parameters of the HMSC model. Effective Sample Size (ESS) over 1000 indicate good fit, while Potential Scale Reduction Factors (PSRF) values of under 1.1 (though ideally 1.01) are considered converged for MCMC sampling.

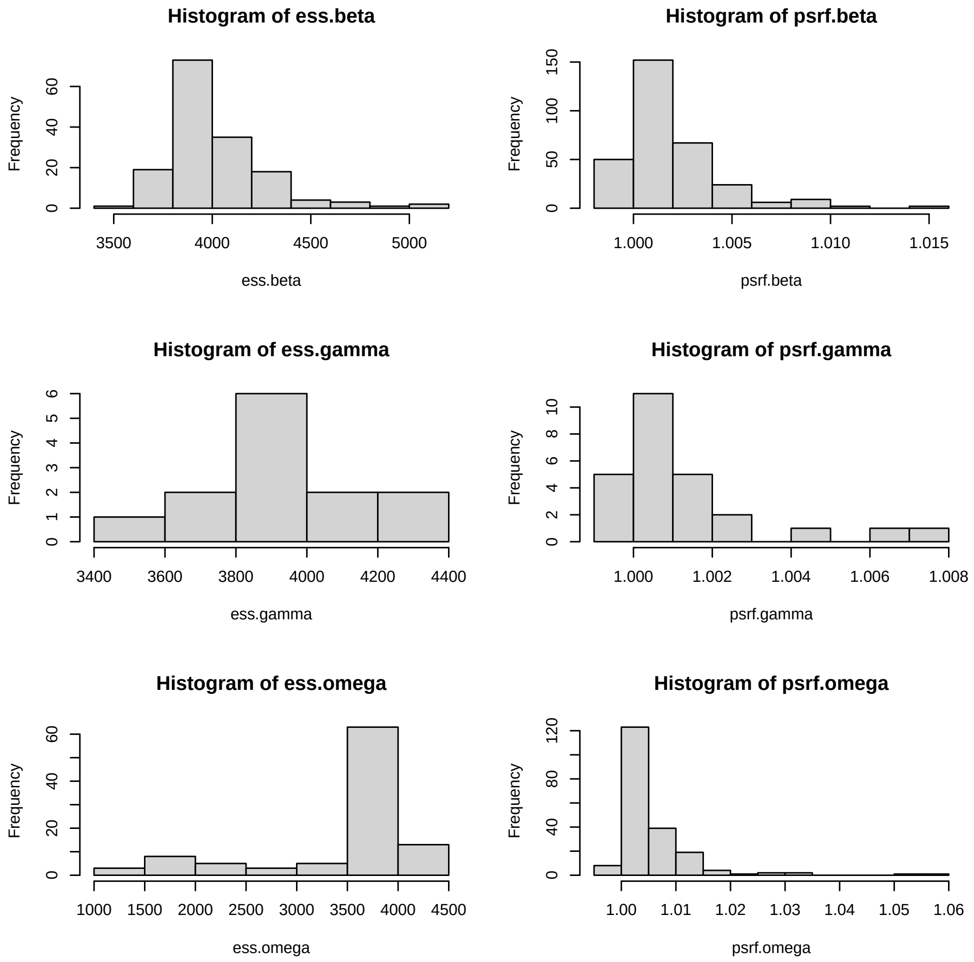

Table S2

Detailed Variance Partitioning results for taxa across different environmental components, including Chemical, Vegetation, Structural, and Random Effects (spatial, season, year). Values represent the proportion of variance explained by each component for the respective taxa.

Taxa	Chemical	Vegetation	Structural	Random: Spatial	Random: Season	Random: Year
Anopheles claviger	0.11	0.07	0.03	0.04	0.00	0.00
Anopheles maculipennis	0.05	0.04	0.03	0.00	0.00	0.00
Culiseta annulata	0.03	0.03	0.01	0.47	0.00	0.00
Culex pipiens	0.03	0.04	0.01	0.38	0.01	0.00
Damselfly larva	0.04	0.06	0.02	0.09	0.03	0.05
Dragonfly larva	0.01	0.02	0.01	0.00	0.00	0.00
Gammarus spp.	0.04	0.06	0.03	0.02	0.01	0.01
Saucerbug	0.01	0.01	0.01	0.00	0.00	0.00
Water beetle	0.02	0.02	0.01	0.05	0.02	0.04
Water beetle larva	0.01	0.02	0.01	0.02	0.00	0.01
Water boatman	0.04	0.03	0.02	0.03	0.01	0.14
Water scorpion	0.01	0.01	0.01	0.00	0.00	0.00
